# Atypical onset of nephropathic infantile cystinosis in a Russian patient with rare *CTNS* mutation

**DOI:** 10.1002/ccr3.1678

**Published:** 2018-08-11

**Authors:** Kozina A. Anastasiya, Okuneva G. Elena, Baryshnikova V. Natalia, Krasnenko Yu. Anna, Tsukanov Yu. Kirill, Klimchuk I. Olesya, Nikishina A. Tatiana, Fedoniuk D. Inessa, Surkova I. Ekaterina, Shatalov A. Peter, Ilinsky V. Valery

**Affiliations:** ^1^ Institute of Biomedical Chemistry Moscow Russia; ^2^ Genotek Ltd. Moscow Russia; ^3^ Pirogov Russian National Research Medical University Moscow Russia; ^4^ Veltischev Research and Clinical Institute for Pediatrics of the Pirogov Russian National Research Medical University Moscow Russia; ^5^ Russian Children's Clinical Hospital Moscow Russia; ^6^ Vavilov Institute of General Genetics Moscow Russia

**Keywords:** *CTNS* gene, cystine, exome sequencing, Fanconi syndrome, nephropathic cystinosis

## Abstract

We report a Russian patient with atypical onset of infantile nephropathic cystinosis. The disease debuted with vomiting and loss of weight and motor skills. Nephropathic changes appeared 6 months after onset of disease. Exome sequencing can be useful for diagnosing cystinosis in patients with neurological abnormalities before onset of nephropathic symptoms.

## 1 INTRODUCTION

Nephropathic cystinosis is a rare autosomal recessive disease. This disease is characterized by accumulation of disulfide amino acid cystine in lysosomes due to a defect in membrane transport protein (encoded by cystinosin gene).^1^ The incidence of cystinosis in general population is 1:100 000‐1:200 000, but more cases were reported in European and US populations.^1^ Three clinical forms of cystinosis are distinguished depending on age of manifestation and severity: nephropathic infantile form (OMIM 219800); juvenile form or intermediate cystinosis (OMIM 219900); and non‐nephropathic adult form or benign non‐nephropathic cystinosis (OMIM 219750).

Infantile cystinosis is the most common (up to 95% of all cases) and the most severe form of the disease. This form develops in the first year of life as Fanconi syndrome (physical developmental delay, rachitic changes in bones, polyuria, polydipsia, hyperphosphaturia, hyperaminoaciduria, glucosuria). Without specific treatment, Fanconi syndrome rapidly progresses to kidney failure at the end of the first decade of life.^1^ The juvenile form of cystinosis is also accompanied by kidney damage, but with manifestation in adolescence.^2^ The adult benign form of cystinosis is manifested only by eye damage (due to deposition of cystine crystals in the cornea) and development of photophobia without signs of damage to other organs.^3^, ^4^ All three forms of disease are caused by mutations in *CTNS* gene (OMIM 606272).^5^

The cystinosin gene, *CTNS*, is located on the short arm of chromosome 17 (locus 17p13).^1^, ^5^, ^6^ The 23‐kb cystinosin gene includes 12 exons. First two exons and the first 19 nucleotides of exon 3 constitute untranslated region.^5^ *CTNS* encodes lysosomal transmembrane protein called cystinosin. It is 367 amino acids long protein that includes seven transmembrane domains and eight potential glycosylation sites. Cystinosin transfers cystine from lysosomes to cytoplasm.^7^

The most common mutation in *CTNS* gene is a large 57‐kb deletion, spanning exons 1‐10. This mutation is homozygous in 50% of patients with cystinosis in North America and Europe.^1^, ^8^ 57‐kb deletion leads to complete absence of protein and probably occurred approximately 1500 years ago in Europe.^9^ To date, 147 mutations in *CTNS* are reported in HGMD database (http://www.hgmd.cf.ac.uk/ac/gene.php?gene=CTNS).

Patients with classical infantile cystinosis generally have deletions or other *CTNS* mutations resulting in complete absence of protein. Patients with intermediate or benign form of cystinosis usually have a severe heterozygous *CTNS* mutation (eg, 57‐kb deletion or nonsense mutation) and another trans‐acting heterozygous mutation (eg, missense mutation), with decreased transport function of cystinosin.^1^, ^8^, ^10^, ^11^

In this study, we analyzed clinical and genetic characteristics of a 2‐year‐old boy with infantile form of cystinosis. The patient developed from nonspecific symptoms (vomiting, weight loss and loss of motor skills) to specific symptoms (glucosuria, proteinuria, cystine crystals in the cornea) within 1 year. Exome sequencing identified a homozygous missense mutation in exon 8 of *CTNS* gene (c.518A>G, p.Tyr173Cys).

## 2 METHODS

### 2.1 Editorial policies and ethical consideration

All research was approved by the ethics committee of Genotek Ltd. (02/2018). The study conforms to the Declaration of Helsinki. The patient's parents have provided written informed consent. The patient's parents gave written informed consent to studies and publication of clinical information, images, and sequencing data.

### 2.2 Case presentation

The patient was a 2‐year‐old boy from Russian republic of Ingushetia. Both parents and younger sibling (female) were asymptomatic. Family history is not burdened. Parents may be related, as the mother of the child and the grandmother (father's line) are from neighboring villages of Malgobek region of Ingushetia.

The patient is the first child of healthy parents. Pregnancy proceeded against the background of gestosis in the first and second trimester, threats of interruption for a period of 12 weeks, anemia, and lack of hydration. The child was born as a result of fast unassisted childbirth. His birth weight was 3570 g and height was 55 cm. Apgar scores were 6/7. Early motor development slightly delays: He attained head holding at 3 months, sitting without support at 6 months, by the year the boy got up and walked with support. From the age of 8 months, the baby had a weight loss of 2 kg for 2 months with episodes of repeated vomiting. At the age of 1 year 2 months against a background of acute respiratory viral infection, there was a regress in development—the boy stopped walking. Subsequently, rachitic skeletal changes, an increase in the volume of the abdomen appeared, and a loss of motor skills progressed. Magnetic resonance imaging (MRI) of brain revealed a mildly expressed leukopathy in white matter of parietal lobes. Proteinuria and glucosuria appeared at the age of 1 year 8 months.

At the age of 2 years 2 months, he was admitted to Russian children's clinical hospital with psychomotor retardation and treatment resistance. On physical examination, he was observed to have a pronounced delay in psychomotor development (he did not get up, walk, crawl; he could only keep his head and turn over). Neurological examination revealed muscular hypotrophy, tendency to hypotension in axial musculature, and uniform decrease in tendon reflexes from hands and feet. MRI of brain revealed subatrophic changes, moderate expansion of the lateral ventricles within the subatrophy with compensated liquorodynamics, and the presence of hypomyelinization zones in the periventricular white matter of the posterolateral regions.

Clinical exome sequencing was carried out by Genotek Ltd. Genomic DNA from peripheral blood sample was extracted using QIAamp DNA Mini Kit (Qiagen, Hilden, Germany). DNA libraries were prepared using NEBNext Ultra DNA Library Prep Kit for Illumina (New England Biolabs, Ipswich, Massachusetts, USA) with adapters for sequencing on Illumina platform. Double barcoding was performed by PCR with NEBNext Multiplex Oligos for Illumina kit. Quality control of DNA libraries was carried out using Bioanalyzer 2100 (Agilent Technologies, Santa Clara, California, USA). We used SureSelect XT2 (Agilent Technologies, Santa Clara, California, USA) for target enrichment. Enriched samples were sequenced on Illumina HiSeq 2500 using pair‐end 100 base pairs reads. After sequencing, we trimmed 3′‐nucleotides with read quality below 10 using Cutadapt. Raw reads were aligned to reference genome hg19 (GRCh37.p13) using BWA MEM. Deduplication of reads was carried out using SAMtools rmdup. FastQC was used for data quality control. We called short variants using GATK HaplotypeCaller according to GATK Best Practices DNA‐seq. The effect of each mutation was assessed using snpEff. To assess pathogenicity and conservatism, the data were extracted from the dbNSFP, ClinVar, OMIM database, and HGMD, as well as using the SIFT and PolyPhen‐2 utilities to predict pathogenicity of the mutation. Information on the frequency of mutations was taken from 1000 Genomes project, ExAC, and Genotek frequency data. Description of mutations and their pathogenicity was predicted according to the Standards and Guidelines developed by ACMG (American College of Medical Genetics and Genomics), AMP (Association for Molecular Pathology), and CAP (College of American Pathologists).^12^ Copy number alterations were determined using CNVkit. *CTNS* variant identified by exome sequencing was confirmed by Sanger sequencing of patient, parental, and sibling DNA samples.

After exome sequencing, on admission in Veltischchev Research and Clinical Institute for Pediatrics, patient was 9 kg in weight and 80 cm in height, with low weight‐for‐height proportion and disharmonious physical development. Deficiency of subcutaneous fat was noted. Attention was paid to rachitic changes in skeleton: expansion of metaphyses, keel‐like deformation of thorax, rachitic “rosary,” varus deformity of lower limbs. Hepatosplenomegaly was noted: liver and spleen protruded from under edge of the costal arch by 2.5 cm. Clinical signs include polyuria and polydipsia (consumed liquid per day 1300 mL, daily diuresis—1300 mL). Blood tests showed mild anemia, hypokalemia, decrease in uric acid, increase in alkaline phosphatase activity, hypophosphatemia, increase in triglycerides, and creatinine level at the lower limit of the norm. The study of equilibrium of acids and bases of blood revealed metabolic acidosis. Test of thyroid gland hormonal profile showed subclinical hypothyroidism. Level of cystine in leukocytes was normal. Glomerular filtration rate (GFR) (97 mL/min) was within the normal range (80‐120 mL/min). Urine test showed phosphaturia, glucosuria, and low molecular weight proteinuria. Cystine was detected in urine test. Ultrasound examination showed hepatosplenomegaly, multiple foci of increased echogenicity in liver, nephromegaly with thickening, and diffuse changes in renal parenchyma. Radiologic examination revealed pronounced osteoporosis, lagging behind bone age. Ophthalmoscopy revealed cystine crystals in the cornea. Thus, the child has signs of infantile form of nephrotic cystinosis with preserved kidney function.

The patient was commenced on cysteamine (Cystagon), initially 50 mg orally four times daily, to increase gradually to 100 mg four times daily (0.8 g/m^2^/d). Also he was commenced on ophthalmic solution Cystadrops (one drop six times daily) and potassium, phosphorus, vitamin D, energotropic agents. After 6‐month therapy, the patient's condition remains stable and serious with slight positive dynamics. The child gained 1 kg in weight and grew by 0.5 cm. Polydipsia/polyuria decreased to 1000‐1200 mL. Hemoglobin and red blood cell levels, and thyroid‐stimulating hormone and T4 levels were normalized. Minimal phenomena of metabolic acidosis persist. Also, filtration function of the kidneys was preserved, and no signs of nephrocalcinosis were detected.

At the last examination, patient had elevated cystine level in the granulocytes (2.32 nmol) and nephrocalcinosis of both kidneys. Cysteamine dose was increased to 1.25 g/m^2^/d.

## 3 RESULTS AND DISCUSSION

**Figure 1:**
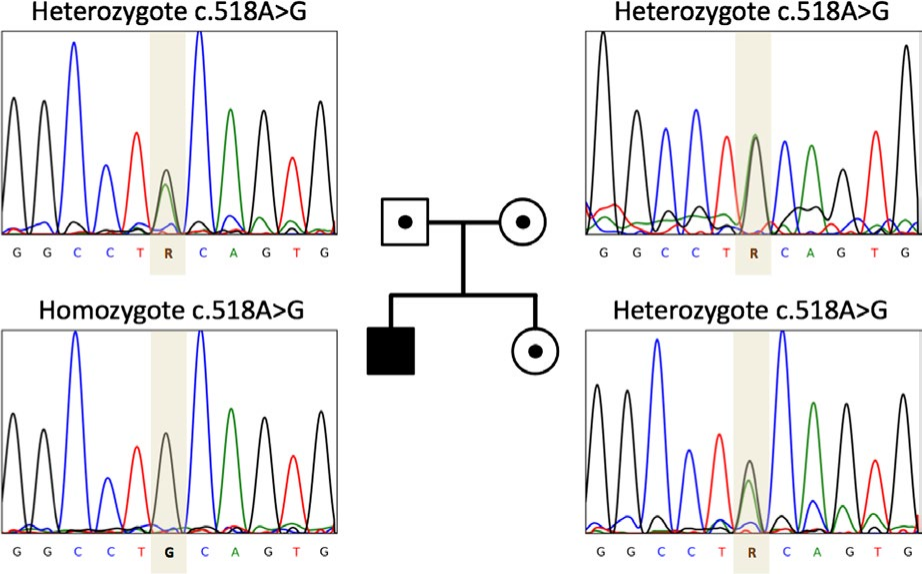
Pedigree and Sanger sequencing details of proband family. Using Sanger sequencing, the inheritance mode of autosomal recessive was confirmed in this family based on identified heterozygote mutation in parents and homozygote in the proband. There are no other family members with cystinosis

Exome sequencing revealed mutation c.518A>G in the 8 exon of the *CTNS* gene in the homozygous state. This mutation caused the replacement of tyrosine by cystine at position 173 in the protein molecule (p.Tyr173Cys). The presence of this homozygous mutation in the *CTNS* gene was confirmed by Sanger sequencing. This mutation is described in the HGMD_record database: CM1110322. We have established the segregation of this mutation in the proband family: The parents are heterozygous for this mutation, and the younger sibling of the proband is also a healthy heterozygous carrier (Figure 1). The mutation c.518A>G (p.Tyr173Cys) found in our patient is classified as pathogenic according to the following ACMG criteria: PS1—the mutation causes an amino acid substitution similar to another known pathogenic mutation; PM2—absent from controls in the frequency databases or present with extremely low frequency; PP3—several independent computer tools predict the pathogenicity of the mutation; and PP5—an authoritative source describes the mutation as pathogenic.^12^ This mutation is not reported in ExAC or in 1000 Genomes Browser. This mutation was not found in our in‐house exomes.

Mutation c.518A>G (p.Tyr173Cys) in the homozygous state was first detected in a patient from Turkey with infantile form of cystinosis and described in the article by Topaloglu et al^13^ Patient was characterized by manifestation of clinical symptoms at the age of 12 months. By the age of 15.5 years, he had proteinuria with preserved kidney function on the background of cysteamine therapy. Thus, our case is the second description of the mutation c.518A>G (p.Tyr173Cys) in the homozygous state as the cause of the infantile nephropathic cystinosis. However, the absence in the article of Topaloglu of a detailed description of symptoms and dynamics of the disease makes it difficult to compare clinical symptoms of Turkish and Russian patients with the mutation c.518A>G (p.Tyr173Cys).

Cystinosis is the main cause of the inherited Fanconi syndrome. Thus, it should be suspected in children of the first 3 years of life with a delay in physical and psychomotor development as well as signs of proximal tubulopathy.

Despite the fact that the accumulation of cystine in the tissues begins in utero, patients with infantile cystinosis are usually born from an uncomplicated pregnancy and have normal anthropometric indicators at birth. Clinical symptoms of the disease are absent at birth and gradually develop in the first months of life. By the age of 6‐12 months, nonspecific symptoms appear, such as delay of physical and motor development, attacks of vomiting, episodes of fever with dehydration.^14^ Kidneys are the first affected organs. Symptoms of Fanconi syndrome (polyuria, polydipsia, dehydration, and acidosis) appear also by the end of the first year of life. In most patients, GFR remains normal up to 2 years and then gradually decreases with the formation of the terminal stage of kidney failure at the end of first decade.^15^ Cystinosis accounts for 5% of childhood renal failure cases.^16^

In this case, detailed clinical characteristics were carried out after detection of pathogenic mutation of the cystinosin (*CTNS*) gene. Thus, molecular diagnosis was confirmed by identification of cystine crystals in the cornea when examined by a slit lamp. Crystals of cystine in the cornea are invariably present in all patients with infantile form of cystinosis after 16 months,^4^ which makes diagnosis at an earlier time difficult. In addition, pediatricians and neuropathologists rarely prescribe an ophthalmoscopic examination of cystine deposits in the cornea in children with delay in psychomotor development even in combination with proteinuria and glucosuria, as described in our case. With increasing availability and reducing costs of genetic tests such as high‐throughput next‐generation sequencing (NGS), it might be feasible to screen pathogenic mutations of *CTNS* gene for individuals with nonspecific clinical symptoms of cystinosis. Differential diagnosis of cystinosis is performed with other hereditary causes of Fanconi syndrome: oculocerebral syndrome (low syndrome), Fanconi‐Bickel syndrome, Dent syndrome (hypophosphatemic rickets), tyrosinemia, Wilson's disease, galactosemia, hereditary fructose intolerance, type I glycogenosis, I‐cell disease, metachromatic leukodystrophy, mitochondrial diseases.

In the described clinical case, the leading symptoms were loss of motor functions and delay in physical development that sent diagnostics to exclude primarily degenerative central nervous system diseases. We need a clinical exome, rather than a separate *CTNS* gene because patient had a wide range of symptoms in the absence of significant clinical and laboratory results for the diagnosis.

The majority of missense mutations in the homozygous and compound heterozygous state lead to a more mild course—intermediate or benign forms of cystinosis.^17^ However, in addition to the type of mutation, it is necessary to consider what the functional significance of the damaged region of the protein is. The mutation c.518A>G (p.Tyr173Cys) apparently affects the gene region responsible for the formation of one of the most active regions of the protein, the second transmembrane domain, which suggests a more severe disruption of its transport function. All described missense mutations for infantile form of cystinosis tend to lie in the transmembrane domains of the protein, or they capture the sites of glycosylation. Another type of mutation is described in the 173 codon of the cystinosin protein is a dinucleotide deletion c.519_520delCA (p.Tyr173Terfs), which leads to the formation of a premature stop codon. The mutation c.519_520delCA (p.Tyr173Terfs) was described as pathogenic and resulted in a severe form of infantile nephropathic cystinosis in the compound heterozygous state with the missense mutation c.1015G>A (p.G339R) in the 12 exon of the *CTNS* gene.^18^ At the same time, missense mutation c.1015G>A (p.G339R), which disrupts the structure 7 of the transmembrane protein domain, in the homozygous state also leads to a severe form of nephropathic cystinosis.^19^ These data may be indirect evidence of our assumption: Missense mutations in conserved transmembrane domains significantly impair protein function, which is comparable in effect to deletions leading to its complete absence. That is, such missense mutations are mutations of the type loss of function in the presence of a protein product.

We did not perform functional studies, but analyzed the possible effect of the amino acid substitution on the structure and function of the cystinosin protein using PolyPhen2 and SIFT. Both methods predict significant impairment of the function or structure of the cystinosin protein. Given that the ethnicity of the patient described in the article by Topaloglu et al^13^ is not indicated, and the disease belongs to rare hereditary diseases, we cannot draw conclusions about the contribution of the mutation to the development of the disease among different ethnic group.

In the present study, we identified a missense *CTNS* mutation, c.518A > G (p.Tyr173Cys), that was associated with severe phenotype of infantile nephropathic cystinosis. This mutation is a rare previously reported pathogenic mutation and has not been previously documented in Russian population. Also, we showed that exome sequencing can be useful for early diagnosing cystinosis in patients with neurological abnormalities before onset of specific nephropathic symptoms. This finding will be helpful for the clinical diagnosis, prenatal diagnosis, and genetic counseling in patients with the same mutation.

## AUTHORSHIP

KAA, OEG, BNV, KAY, TKY, KOI, NTA, FID, SEI, SPA, and IVV: met the International Committee of Medical Journal Editors (ICMJE) criteria for authorship. KAA, OEG, BNV, SEI, and SPA: contributed to data collection and the first draft of the manuscript. KAY, TKY, and KOI: carried out the mutation analysis. NTA and FID: cared for the patient. IVV: was a mentor who contributed equally to this work. All authors read and approved the final manuscript.

## CONFLICT OF INTEREST

KAA, OEG, BNV, KAY, TKY, KOI, SEI, SPA, IVV are employed by Genotek Ltd. The authors declare that they have no other competing interests.
